# Shortening the Saturation Time of PBAT Sheet Foaming via the Pre-Introducing of Microporous Structures

**DOI:** 10.3390/ma18051044

**Published:** 2025-02-26

**Authors:** Fangwei Tian, Junjie Jiang, Yaozong Li, Hanyi Huang, Yushu Wang, Ziwei Qin, Wentao Zhai

**Affiliations:** School of Materials Science and Engineering, Sun Yat-sen University, Guangzhou 510275, China; tianfw@mail2.sysu.edu.cn (F.T.); jiangjj37@mail.sysu.edu.cn (J.J.); liyz33@mail2.sysu.edu.cn (Y.L.); huanghy285@mail2.sysu.edu.cn (H.H.); wangysh56@mail2.sysu.edu.cn (Y.W.); qinzw3@mail2.sysu.edu.cn (Z.Q.)

**Keywords:** PBAT foam, pretreatment, CO_2_ diffusion, high expansion ratio, high resilience

## Abstract

Poly (butylene adipate-co-terephthalate) (PBAT) foam sheets prepared by foaming supercritical fluids are characterized by high resilience, homogeneous cellular structure, and well-defined biodegradability. However, the inert chemical structure and the rigid hard segments restrict the diffusion of CO_2_ within the PBAT matrix, resulting in extremely long gas saturation times as long as 9 h at a thickness of 12 mm. In this study, microporous structures were pre-introduced into the PBAT matrix to provide a fast gas diffusion pathway during the saturation process. After 2 h of saturation, PBAT foam sheets with expansion ratio of 10 to 13.8 times were prepared. The interaction of CO_2_ with PBAT was systematically investigated, and the CO_2_ sorption process was evaluated kinetically and thermodynamically using the Fickian diffusion theory. The solubility and diffusion rate of CO_2_ in pretreated PBAT sheets with different microporous sizes and densities were investigated, and the effects of pretreatment strategies on the foaming behavior and cell structure of PBAT foam sheets were discussed. The introduction of a microporous structure not only reduces saturation time but also enhances solubility, enabling the successful preparation of soft foams with high expansion ratios and resilience. After undergoing foaming treatment, the PBAT pretreated sheets with a 10 μm microporous structure and a density of 0.45 g/cm^3^ demonstrated improved mechanical properties: their hardness decreased to 35 C while resilience increased to 58%, reflecting enhanced elastic recovery capabilities. The pretreatment method, which increases the diffusion rate of CO_2_ in PBAT sheets, offers a straightforward approach that provides valuable insights into achieving rapid and efficient foaming of thick PBAT sheets in industrial applications.

## 1. Introduction

With the progress of time and technological advancements, the demand for high- performance and lightweight materials in the fields of production and daily life is steadily increasing. Microcellular foaming technology is gaining popularity as an environmentally friendly solution for achieving lightweight materials [1,2,3,4]. In this technology, physical blowing agents such as CO_2_ and N_2_ are employed to produce foams with a fine and uniform cellular structure, offering advantages such as high toughness, impact resistance, sound insulation, and thermal insulation [5,6,7]. Furthermore, the microcellular foaming process can accommodate the customization of foam materials, enabling the production of bimodal foam, open-cell foam, and folded-wall foam, among others, through process regulation [8,9,10]. Currently, the application of microcellular foaming technology has successfully facilitated the lightweight preparation of a variety of polymer materials, including general-purpose plastics such as polypropylene (PP) [11], polyethylene (PE) [12], and polystyrene (PS) [13], as well as thermoplastic elastomers such as thermoplastic polyurethane (TPU) [14] and thermoplastic polyester elastomers (TPEE) [15]. The technology also extends to engineering plastics, including polycarbonate (PC) [16], polyphenylene oxide (PPO) [17], and polyetherimide (PEI) [18].

Recently, the research on biodegradable polymer foams has garnered significant attention due to their environmentally friendly properties [19,20,21,22]. Among them, polybutylene adipate-co-terephthalate (PBAT), a biodegradable aliphatic-aromatic copolyester, typically consists of 42–48% butylene terephthalate (BT) units and 52–58% butylene adipate (BA) units [23,24]. PBAT exhibits high ductility and flexibility comparable to traditional polyolefins, as well as low cost and excellent processing performance, mechanical properties, and gas barrier properties [25,26,27]. It has been widely applied in shopping bags, packaging films, and agricultural mulching films [28,29,30]. Regarding the lightweighting of PBAT, significant progress has been made by researchers. Wang et al. [31] systematically investigated the interaction between the physical blowing agents CO_2_ and N_2_ with PBAT, successfully producing PBAT foams with high expansion ratios and low shrinkage using a gas mixture as the blowing agent. Zhang et al. [24] analyzed the effect of PBAT’s chain segment composition on its foaming performance, showing that an increase in BT segment content led to higher crystallinity, elevated foaming temperatures, and a narrower foaming temperature range. When the BT content reached 52%, PBAT exhibited optimal foaming performance and biodegradability. Moreover, our previous research demonstrated that incorporating small amounts of poly(butylene succinate) (PBS) modification, combined with co-blowing agents, can successfully produce high-resilience, heat-resistant soft PBAT foams [32]. Additionally, microcellular foaming technology has been shown to successfully foam PBAT beads, which can then be molded into complex three-dimensional parts [33,34]. As an environmentally friendly polymer, PBAT demonstrates excellent foaming performance in lightweight manufacturing and holds significant commercial potential for applications in buffer materials, electromagnetic shielding, and strain-sensing materials in the future [35,36,37,38].

It is important to note that in the current PBAT foaming studies, the PBAT substrates are limited to particles, rods, and thin sheets with a thickness not exceeding 3 mm. In these situations, the influences of gas diffusion and adsorption time on the thin PBAT samples are minimal. However, this limitation has kept PBAT foaming research confined to the laboratory stage, preventing commercialization and broader application. Since the polymer foaming process involves gas diffusion and adsorption mass transfer, the factors influencing the diffusion rate (diffusion coefficient) are complex [39,40]. Firstly, the stiffness of the molecular chain significantly affects gas diffusion. In their early studies on high-performance microcellular polysulfone foams, Sun and Mark [41] found that higher crystallinity hinders gas diffusion. Later, Huang et al. [42] examined the influence of the ratio of rigid and flexible segments in PU on gas barrier properties and observed that an increased proportion of rigid segments significantly enhances the gas barrier properties of PU. This finding aligns with the free volume theory, which suggests that an increase in rigid segments reduces the free volume between molecular chains, decreasing the number of “holes” and thus reducing gas diffusion [43,44]. Secondly, variations in temperature and pressure also influence gas diffusion. As temperature increases, the mobility of molecular chains and gas molecules strengthens, resulting in a larger diffusion coefficient. Higher pressure, on the other hand, creates a greater concentration gradient during diffusion, which also increases the diffusion coefficient. Schnitzler et al. [45] provided an explanation for this in their study of CO_2_ mass transfer kinetics in PET. They found that the glass transition temperature serves as a critical point: when the adsorption temperature exceeds this threshold, the adsorption capacity of CO_2_ significantly increases, and the lower viscosity of CO_2_ further enhances its diffusion rate.

To improve the gas diffusion rate and efficiently produce high-performance foam, several researchers have made significant efforts. Sun et al. [46] employed the “partitioning method” to create internal tunnels in the PEI substrate, effectively dividing the original large substrate (millimeter scale) into numerous smaller parts (micrometer scale). These interpenetrating pores allowed for rapid CO_2_ diffusion and increased the adsorption capacity, which reached 9.18 wt% at 35 °C in just 1 h, significantly higher than the 4.36 wt% of non-porous PEI. Gunasekaran et al. [47] proposed a process for preparing TPU graded porous foam structures using 3D printing combined with subcritical CO_2_ foaming. This process creates a stacked structure with spatial pores by controlling the filling density, providing channels for rapid gas molecule passage and ensuring quick saturation. Chen et al. [48] explored the in situ melting and rheological behavior of PP under high-pressure conditions and introduced a dynamic variable-pressure saturation strategy. This method uses periodic pressure variations to enhance gas diffusion, ultimately enabling low-temperature foaming to produce high-expansion-ratio foams. However, these designs are relatively complex and require high equipment precision, which limits their potential for efficient mass production in industrial settings. Furthermore, these foaming studies still focus on small-scale samples, which may not be representative and could face challenges in broader application or generalization.

Herein, to bridge the technical gaps in sustainable polymer foam fabrication, the main objective of this study is to evaluate and optimize the CO_2_ adsorption process in the PBAT matrix through both kinetic and thermodynamic analysis, using Fickian diffusion theory. Additionally, this work aims to propose an efficient short-time foaming method for large-scale PBAT production. By introducing a microporous structure via batch micro-foaming, the study seeks to achieve rapid and graded CO_2_ diffusion by controlling wall thicknesses between micropores. The research systematically investigates the solubility and diffusion behavior of CO_2_, alongside the foaming characteristics of pretreated PBAT sheets with varying microporous structures. The findings highlight the potential of these pretreated sheets to produce high-expansion foams with superior performance, offering new perspectives for developing environmentally friendly, high-performance foams.

## 2. Materials and Methods

### 2.1. Materials

Poly(butylene adipate-co-terephthalate) (PBAT, 2208) raw resin was obtained from Jinghui Zhaolong High-tech Co., Ltd. (Taiyuan, China), with a density of 1.24 g/cm^3^ and a melt mass-flow rate (MFR) and carboxyl content of 4.69 g/10 min and 12.35 Mol/t, respectively. The carbon dioxide (CO_2_) with a purity of 99.9% was purchased from Guangzhou Guangqi Gas Co., Ltd. (Guangzhou, China), and it was used as the physical blowing agent.

### 2.2. Sample Preparation

The PBAT pellets were initially dried in a blast oven at 80 °C for 5 h to ensure complete moisture removal. Subsequently, the pellets were injection molded into sheets with dimensions of 10 cm by 5 cm (length by width) at 12 MPa and 155 °C, using an injection molding machine (Guangzhou POTOP Co., Ltd., Guangzhou, China). The sheet thicknesses were 1 mm, 3 mm, 6 mm, and 12 mm. Notably, the length and width of the PBAT sheets were much larger than the thickness to ensure that gas diffusion during the saturation process was predominantly influenced by the thickness direction. Following injection molding, the PBAT sheets were prepared for subsequent use in various foaming strategies.

**Figure 1:**
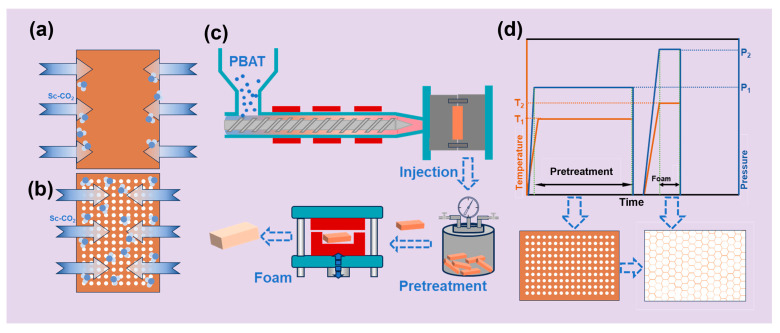
Schematic of PBAT foam preparation. (**a**) One-step foaming of PBAT sheets; (**b**) foaming of PBAT pretreated sheets; (**c**) schematic diagram of the PBAT sheet pretreatment–short-duration foaming process; (**d**) parameter distributions during pretreatment–short-duration saturated foaming process.

As illustrated in Figure 1, the fabrication of PBAT foamed sheets employs two distinct strategies. The conventional one-step approach (Figure 1a) enables direct production of large-volume foamed sheets through single-stage processing. In this method, individual PBAT sheets undergo complete CO_2_ saturation within a mold-foaming machine prior to instantaneous foaming. In contrast, Figure 1b–d present an innovative two-stage strategy involving batch pretreatment followed by short-duration secondary foaming. This advanced protocol begins with batch saturation, in which multiple PBAT sheets are simultaneously pressurized with CO_2_ in an autoclave. Subsequent rapid pressure release induces controlled formation of microporous structures while maintaining dimensional stability (<2% size variation). The pretreated PBAT sheets are then transferred to temperature-controlled mold-foaming equipment, where precision thermal regulation based on saturation parameters ensures optimal gas diffusion during CO_2_ injection. Final rapid depressurization at the designated foaming temperature yields high-volume PBAT foams with uniform cellular architectures.

### 2.3. Characterization

The sorption of supercritical CO_2_ in polymers significantly influences gas diffusion behavior and the formation of cellular structures. The convective diffusion equation serves as the fundamental equation for characterizing the mass transfer dynamics of a fluid system. This process is governed by Fick’s second law of diffusion:

(1) $$\frac{\partial C}{\partial t}=D\frac{\partial^{2}C}{\partial x^{2}}$$

where C is the concentration of CO_2_, D is the diffusion coefficient (m^2^/s), and x is the position [49]. Equation (1) can be derived under the assumption that changes in thickness due to CO_2_ plasticization and swelling are neglected and that the gas diffusion process occurs only in the one-dimensional direction (the thickness direction). Crank [50] derived the relationship between the change in sample mass and the diffusion coefficient of the sample during the diffusion process, as expressed in the following equation:

(2) $$\frac{M_{t}}{M_{\infty }}=1-\frac{8}{\pi^{2}}\sum_{n=0}^{\infty }\frac{1}{{\left(2n+1\right)}^{2}}exp\left[\frac{-{\left(2n+1\right)}^{2}\pi^{2}Dt}{4L^{2}}\right]$$

where $M_{t}$ and $M_{\infty }$ are the mass of the sample obtained at moment t and dissolution equilibrium, respectively, t is the sorption time (s), and L is the sample thickness (mm).

For longer diffusion times or when in the saturated state, Equation (2) can be simplified to the following:

(3) $$\frac{M_{t}}{M_{\infty }}=1-\frac{8}{\pi^{2}}exp\left[\frac{-\pi^{2}Dt}{L^{2}}\right]$$

Equation (3) can be applied to both sorption and desorption processes [51]. The relationship between $ln\;(1-M_{t}/M_{\infty })$ and t can be obtained through linear fitting, and the diffusion coefficient can be calculated from the slope and the initial thickness.

During the test, PBAT samples were removed from the container after saturation was complete, with a 30 s interval between sample removal and weight measurement. The weight of the sample over time was measured on a 1 in 100,000 precision balance (AUW120D, Shimadzu, Kyoto, Japan), and the ambient temperature and humidity were 22 °C and 70%, respectively. Sample tests were repeated three times and then the mean and standard deviation were calculated to ensure experimental accuracy and to provide feedback on errors.

The thermal properties of PBAT foamed sheets were characterized using differential scanning calorimetry (DSC 250, TA Instruments, New Castle, DE, USA) over a temperature range from 25 to 180 °C with a heating/cooling rate of 10 °C/min. The sample’s degree of crystallinity ($X_{c}$) can be calculated as follows:

(4) $$X_{c}=\frac{∆H_{m}}{∆H_{m0}}\times 100\%$$

where $∆H_{m}$ is the enthalpy of melting (J/g), and $∆H_{m0}$ is the theoretical enthalpy of the 100% crystalline PBAT (114 J/g) [52]. The mean and standard deviation were calculated after repeating the test three times for the same sample to ensure experimental accuracy and to provide feedback on the errors.

The density of the foamed samples was obtained with a density balance (DA-300 M, DahoMeter, Dongguan, China). The expansion ratio (φ) of ETPU beads was calculated by the following equation:

(5) $$\varphi =\frac{\rho_{s}}{\rho_{f}}$$

where $\rho_{s}$ and $\rho_{f}$ are the densities of the solid PBAT samples and PBAT foamed sheets, respectively (g/cm^3^) [53]. Due to the shrinkage and recovery behavior of PBAT foams, the expansion ratio was measured immediately at the end of foaming and recorded as the initial expansion ratio ($\varphi_{initial}$) and then measured again after 10 days of stabilization and recorded as the final expansion ratio ($\varphi_{final}$).

Scanning electron microscopy (EM-30, COXEM, Daejeon, Republic of Korea) was used to observe the cellular morphology of PBAT foam. A sharp blade was used to make a quick cut from the middle of the PBAT foam sheet, and the surface of the section was sputtered with gold before being observed using SEM. Cellular structural information, including cell density ($N_{n}$), average cell size (d), and cell wall thickness (δ), was measured by counting at least 100 cells using Nano Measurer 1.2 software. The cell density was calculated using the following equation:

(6) Nn=nA23φ

where $N_{n}$ is the cell density, *n* is the number of cells in a micrograph, A is the area of the micrograph, and φ is the expansion ratio calculated as above [54].

On the other hand, the cell mean wall thickness was evaluated through Equation (7):

(7) $$\delta =d\left(1/\sqrt{1-\rho_{f}/\rho_{s}}\right)-1$$

where δ is the cell wall thickness (μm), d is the average cell size (μm), $\rho_{s}$ is the density of the solid PBAT sample (g/cm^3^), and $\rho_{f}$ is the density of the PBAT foam (g/cm^3^) [55].

A universal testing machine was used to characterize the compression properties of PBAT foam sheets, which were cut to a uniform size of 5 × 5 × 1 cm. The tests were performed at room temperature, with a compression rate of 10 mm/min and a maximum compression strain of 50%.

The shore C hardness of the PBAT foam was measured by a hardness tester (LX-C, Hongri Instrument Equipment Co., Ltd., Fuding, China). The rebound was tested using a digital-display falling-ball resilience tester from Hongri Instrument Equipment Co., Ltd. Sample tests were repeated three times and then the mean and standard deviation were calculated to ensure experimental accuracy and to provide feedback on errors.

## 3. Results and Discussion

### 3.1. CO_2_ Solubility and Diffusion Coefficient

**Figure 2:**
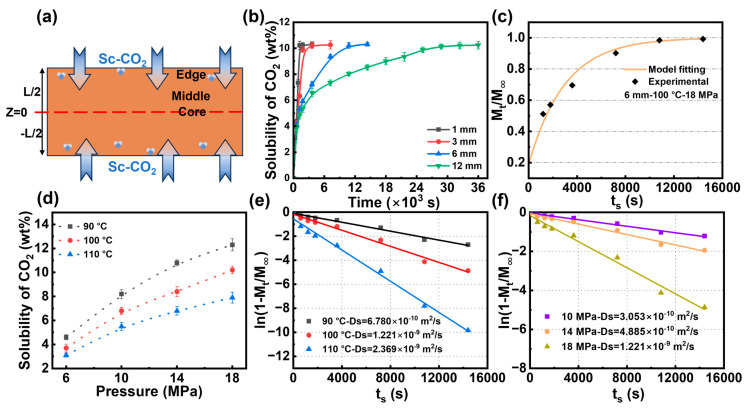
CO_2_ sorption and diffusion in untreated PBAT sheets. (**a**) Schematic diagram of CO_2_ diffusion in PBAT matrix; (**b**) CO_2_ sorption in PBAT sheets of different thicknesses at 100 °C—18 MPa; (**c**) fitting of Fick’s diffusion model; (**d**) isothermal sorption of CO_2_ in PBAT; (**e**) kinetic linear fitting of diffusion coefficients at different temperatures; and (**f**) kinetic linear fitting of diffusion coefficients at different pressures.

The diffusion rate and solubility of gases during the polymer foaming process significantly influence the density and pore structure of foams. A comprehensive study of the solubility and sorption behaviors of CO_2_ in the PBAT matrix is essential for precisely controlling the foaming process of PBAT. The CO_2_ diffusion process within the untreated PBAT substrate is depicted in Figure 2a. In the CO_2_/PBAT binary system, the gas gradually diffuses from the edge of the PBAT sheet toward the core layer, eventually reaching a concentration equilibrium and forming a stable system within the PBAT. Figure 2b illustrates the solubility of CO_2_ vs. saturation time curves in PBAT sheets of different thicknesses. For PBAT sheets with a thickness of 3 mm or less, CO_2_ diffusion reaches saturation within 30 min. However, for sheets with a thickness of 6 mm, CO_2_ solubility increases more rapidly during the first 20 min, after which the process slows and the diffusion becomes more challenging. This trend is even more pronounced in PBAT sheets with a thickness of 12 mm, where equilibrium is reached after 9 h. These results suggest that PBAT is best suited for foaming thin membranes or sheets, and efficiently producing foamed PBAT thick sheets is particularly challenging. In this study, a 6 mm thick sample was selected to investigate CO_2_ diffusion behavior in untreated PBAT sheets, taking into account the effect of saturation time.

Figure 2c shows the CO_2_ sorption content versus sorption time for a 6 mm thick PBAT sheet at 100 °C and a saturation pressure of 18 MPa, with the solid line indicating the fitting results. The fitted data closely align with the experimental data, indicating a high applicability of Equation (3) at long saturation times. Figure 2d displays the isothermal adsorption curves of PBAT at temperatures ranging from 90 °C to 110 °C and pressures from 10 MPa to 18 MPa. The highest CO_2_ sorption content is observed at low temperatures and high pressures, reaching 12.3 wt% at 90 °C and 18 MPa. As temperature increases and pressure decreases, CO_2_ sorption decreases, with a minimum solubility of 3.1 wt% at 110 °C and 6 MPa. These trends are expected: at low temperatures and high pressures, carbon dioxide readily condenses and is easily trapped in the polymer matrix. The solubility of CO_2_ in the PBAT matrix is significantly reduced under combined high-temperature and low-pressure conditions. This phenomenon is primarily attributed to two synergistic factors: first, the increased mobility of both gas molecules and polymer chains at elevated temperatures, which weakens gas–polymer interactions, and second, the reduced gas concentration gradient at lower pressures, which diminishes the driving force for gas dissolution and promotes gas desorption from the polymer matrix. Across all conditions studied, while CO_2_ solubility increases with pressure, it gradually deviates from a linear positive correlation with Henry’s law. This phenomenon has also been discussed by Durrill and Lundberg et al. [56,57], who propose that Henry’s law must be adjusted in high-pressure environments. They argue that high-pressure fluids influence the rearrangement of the polymer molecular chain structure, potentially resulting in the formation or elimination of interchain voids.

The diffusion coefficient (Ds) is a kinetic parameter that reflects the movement of gas molecules within the polymer. Sorption kinetics can be determined from desorption data by incrementally varying the adsorption time. Linear fits of the effects of temperature and pressure on the sorption diffusion coefficient are presented in Figure 2e,f, respectively. In Figure 2e, where the saturation pressure is fixed at 18 MPa, the slope of the curve is observed to gradually increase with rising temperature. When the saturation temperature was increased from 90 °C to 110 °C, the diffusion coefficient rose from 6.78 × 10^−10^ m^2^/s to 2.37 × 10^−9^ m^2^/s, representing a 3.5-fold increase. This suggests that higher temperatures enhance the diffusion rate of the gas. According to dynamic free-volume theory, the diffusion process occurs as gas molecules move through the free-volume pores within the polymer matrix, with the frequency of these molecular jumps influencing the diffusion coefficient [44]. As temperature increases, the segmental mobility of PBAT chains enhances, promoting the formation of transient gaps between the chains. This facilitates the rapid movement of CO_2_ molecules, thereby improving the diffusion coefficient. As shown in Figure 2f, the effect of pressure on the diffusion coefficient is similar to that of temperature. The diffusion coefficient can be increased by a factor of 4 when the pressure is increased from 10 MPa to 18 MPa at a fixed saturation temperature of 100 °C. In a vessel of the same volume, an augmentation in the pressure of CO_2_ implies an escalation in gas concentration, which results in a notable increase in the concentration gradient from the edge of the PBAT matrix to the core, thereby enhancing the gas diffusion rate.

### 3.2. Thermal Behavior of PBAT Under CO_2_

**Figure 3:**
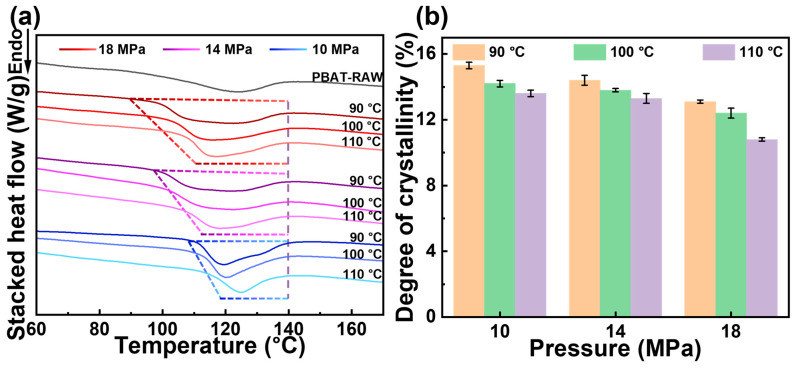
PBAT foam thermal behavior. (**a**) DSC curves of the PBAT samples treated under various pressures and temperatures; (**b**) degree of crystallinity based on DSC pattern.

As a semi-crystalline polymer, the crystallization and thermal behavior of PBAT are closely linked to the gas diffusion process. The DSC results for PBAT foams prepared under varying saturation temperatures and pressures are shown in Figure 3a. The raw PBAT materials exhibit a broad melt peak. However, after saturation foaming, the onset melt temperature of the sample shifts to higher temperatures, and the width of the melt peak narrows. Notably, an increase in temperature or pressure causes the starting melt temperature of the foam sample to decrease, accompanied by a gradual broadening of the melt peak. This behavior is attributed to the decentralized distribution of soft and hard segment chains. On the one hand, temperature- and CO_2_-induced crystallization promotes melt recrystallization during the saturation process, resulting in a more refined and compact crystalline structure, which is reflected by a higher onset melt temperature and a narrower melt peak. On the other hand, elevated temperature and pressure increase plasticization, significantly enhancing molecular chain mobility and disrupting weak hydrogen bonds within the pre-existing crystal structure [38]. This disruption leads to the formation of numerous microcrystals, which in turn broadens the melt peak. Crystallinity data in Figure 3b and Table 1 further reveal that high temperature and pressure cause a marked reduction in PBAT crystallinity (from 15.3% at 90°C, 10 MPa to 10.8% at 110°C, 18 MPa). This phenomenon is attributed to the decrease in the number of crystal regions resulting from the transition from a perfect to an imperfect crystalline structure. The reduction in crystal regions weakens the CO_2_ diffusion barrier, explaining the higher CO_2_ diffusion rate in the PBAT matrix at elevated temperatures and pressures.

### 3.3. Foaming Behavior of PBAT Sheets

**Figure 4:**
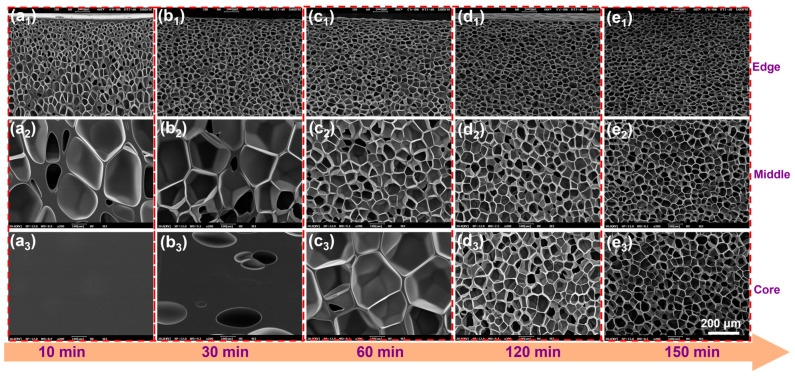
SEM image of the evolution of the cell structure of untreated PBAT sheet (6 mm thickness) with saturation time at 100 °C—18 MPa. (**a_1_**–**e_1_**) evolution of the cell structure in the edge region with saturation time (10–150 min); (**a_2_**–**e_2_**) evolution of the cell structure in the middle region with saturation time (10–150 min); and (**a_3_**–**e_3_**) evolution of the cell structure in the core region with saturation time (10–150 min).

The study of the dynamic relationship between the gas diffusion process and the cell structure provides a foundation for designing the cell structure in the subsequent PBAT pretreatment process. The SEM micrographs in Figure 4 illustrate the evolution of the PBAT cell structure (6 mm in thickness) during the gradual diffusion and CO_2_ saturation at 100 °C and 18 MPa. After 10 min of saturation, PBAT exhibits a gradient cell structure from the edge to the core. Small circular cells are present at the edges, large polygonal cells dominate the middle region, and the core layer shows no cell formation. After 30 min of saturation, the core layer begins to develop cell structures, indicating that the gas has fully penetrated the PBAT matrix within this time frame. As saturation time increases further, the cells at the edges cease to change significantly, while those in the middle and core layers gradually become smaller. After 150 min of saturation, the cells in the middle and core layers adopt a circular shape, consistent with those at the edges. This observation suggests a delayed gas diffusion process, where the edge region reaches gas saturation quickly, the middle region transitions with delayed saturation, and the core layer eventually reaches full saturation.

**Figure 5:**
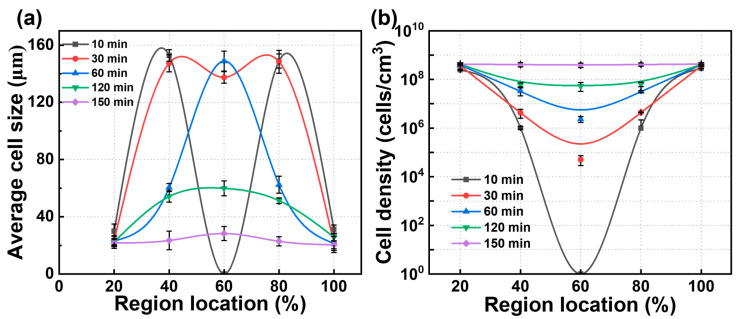
Dynamic information on the cell structure of PBAT foams obtained at 100 °C—18 MPa. (**a**) Cell size across different positions at different times; (**b**) cell density across different positions at different times.

To facilitate the analysis of cell statistics, the PBAT matrix was divided into several regions: the edge region (the first 20%), the middle region (20–40%), the core layer (40–60%), and the symmetric regions (60–80% and 80–100%). The statistical results are presented in Figure 5. Over time, CO_2_ diffusion within the PBAT matrix gradually saturated. As saturation occurred, cell size decreased, and cell density increased in the fully saturated sample compared to the insufficiently saturated one. During the saturation process, which lasted from 10 min to 150 min, the cell size in the edge region decreased from 30 μm to 22 μm within the first 30 min and then stabilized. In the middle region, cell size gradually decreased from 153 μm to 22 μm, but only after saturation time reached 150 min. In the core region, cell size transitioned from a non-porous state to a large size of approximately 150 μm, before gradually decreasing to around 23 μm. Cell density exhibited a similar trend due to gas diffusion: the edge region stabilized first at 4 × 10^8^ cells/cm^3^, while cell density in the middle and core regions gradually increased, aligning with the edge region. Overall, in PBAT sheets, the cell size and cell density in different regions reach steady state at different stages. However, the edge region reaches steady state much faster than the middle and core regions, which is attributed to the rapid diffusion of gases. Therefore, designing and constructing multi-scale “edge regions” can help shorten saturation time.

### 3.4. Pretreatment of PBAT Sheets and Sorption Behavior of CO_2_

**Figure 6:**
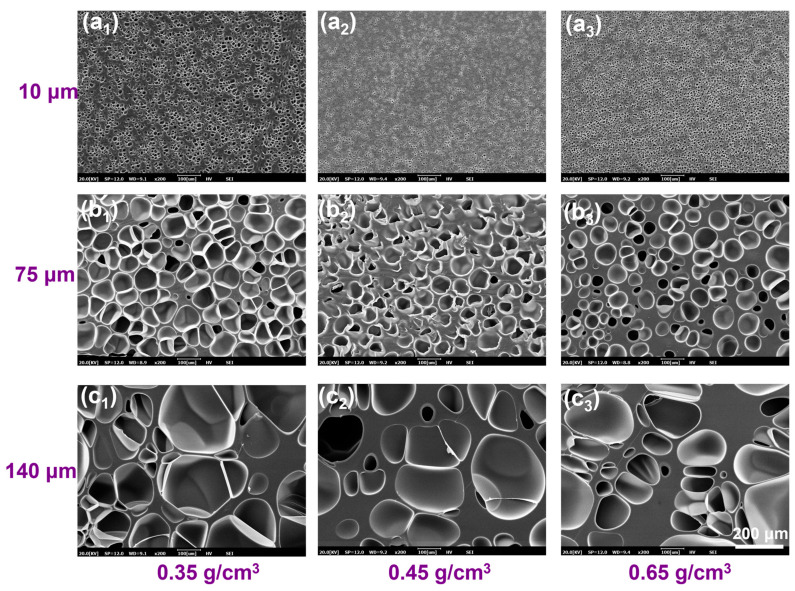
SEM image of microporous structures of typical PBAT pretreated sheets. (**a_1_**–**c_1_**) samples with different microporous sizes at a density of 0.35 g/cm^3^; (**a_2_**–**c_2_**) samples with different microporous sizes at a density of 0.45 g/cm^3^; (**a_3_**–**c_3_**) samples with different microporous sizes at a density of 0.65 g/cm^3^.

**Figure 7:**
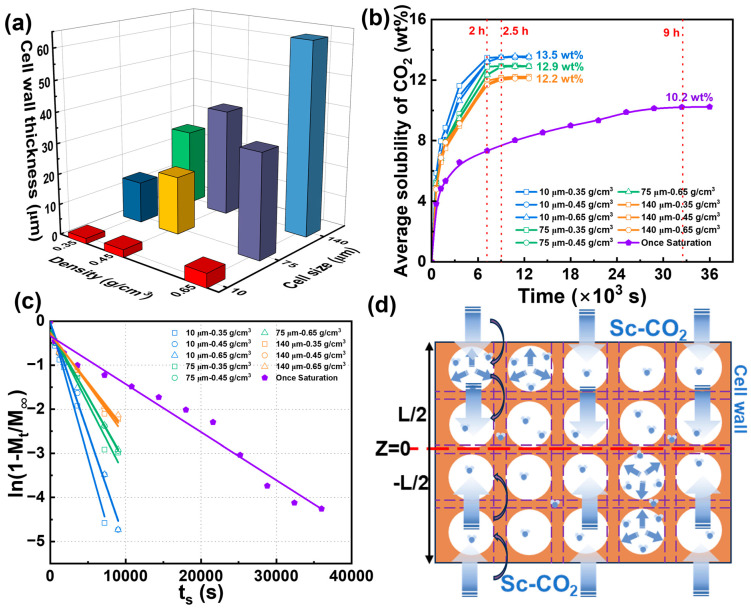
Diffusive behavior of CO_2_ in pretreated PBAT sheets. (**a**) Effect of microporous structure on cell wall; (**b**) CO_2_ sorption process under different microporous structures; (**c**) linear fitting of diffusion coefficients; (**d**) schematic diagram of rapid CO_2_ diffusion in pretreated PBAT sheets.

The pretreatment strategy introduces microcellular structures into the PBAT sheet matrix, creating numerous artificial “edge regions” to reduce saturation time and enhance foaming efficiency. To visualize the role of this pretreatment in the semi-solid batch foaming process, a representative 12 mm thick PBAT sheet was selected as the pretreatment carrier. To reach full saturation, the pretreatment time was set to 12 h. As shown in Table 2, the size of the introduced micropores was controlled to approximately 10 μm at a pretreatment pressure of 18 MPa. As the pressure decreased to 12 MPa and 8 MPa, the micropores expanded to about 75 μm and 140 μm, respectively. Subsequently, the pretreatment temperature was adjusted to prepare PBAT sheets with varying densities of 0.35, 0.45, and 0.65 g/cm^3^. The cross-sectional microporous morphology of PBAT pretreated sheets with different microporous sizes and densities is shown in Figure 6. Combined with Table 2, the micropore density decreases as the micropore size increases. For sheets with a micropore size of 10 μm, the micropore density is on the order of 10^8^–10^9^, while for sheets with a 75 μm micropore size, the density is on the order of 10^6^. The lowest micropore density occurs after pretreatment at 8 MPa, with a density on the order of 10^5^–10^6^. The introduced microporous structure fully occupies the PBAT matrix, enabling rapid diffusion and sorption of CO_2_. As shown in Figure 7a, the thickness of the microporous wall can be indirectly regulated by controlling the density and microporous size of the pretreated sheet. The PBAT pretreated sheet with a microporous size of 10 μm and a density of 0.35 g/cm^3^ has the thinnest microporous wall with a thickness of 1.8 μm. As the micropore size and sheet density increase, the microporous wall thickness also increases, reaching a maximum of 61 μm for the sheet with a micropore size of 140 μm and a density of 0.65 g/cm^3^.

The saturation condition of 100 °C and 18 MPa was selected to further investigate the CO_2_ gas diffusion behavior in PBAT pretreated sheets, with the results shown in Figure 7b. The 12 mm thick untreated PBAT sheet exhibited a strong barrier effect on the gas, requiring 9 h to reach saturation, with a gas solubility of only 10.2 wt%. In contrast, the pretreated PBAT sheets reached full saturation within 2.5 h, and their CO_2_ solubility at saturation was significantly higher than that of the untreated sheet. For the pretreated sheets with a micropore size of 10 μm, the shortest saturation time and highest solubility were observed—2 h and 13.5 wt%, respectively. It can also be seen that as the micropore size increased, the saturation time of the pretreated PBAT sheets slightly lengthened and their solubility gradually decreased. This phenomenon may be attributed to the decrease in microporous density and the increase in the wall thickness between the micropores, which occur as the size of the micropores increases. The increase in the solid portion of the PBAT matrix creates a barrier to gas diffusion, while the reduction in the number of micropores may also decrease the available storage space for CO_2._

The diffusion coefficients of CO_2_ in different pretreated sheets are shown in Figure 7c by linear fitting. Compared to the once-saturated foaming process of untreated PBAT sheets, the diffusion fitting curves of the pretreated sheets shift downward, indicating that the introduction of microcellular structures in the PBAT matrix enhances the diffusion coefficient. As shown in Table 3, the diffusion coefficient is highest when the microporous wall is thinnest, at 2.01 × 10^−8^ m^2^/s, and lowest when the microporous wall is thickest, at 7.16 × 10^−9^ m^2^/s. After pretreatment, the diffusion coefficient increases by a factor of 6 to 16 compared to the single foaming process. A potential explanation for the rapid diffusion process is provided in Figure 7d, where the introduction of microporous structures into the PBAT matrix via pretreatment results in the formation of numerous hollow regions. During diffusion, CO_2_ is first sorbed into the micropore walls, then rapidly fills and saturates the microporous regions. In contrast, untreated PBAT sheets require CO_2_ to diffuse through the entire matrix during saturation, whereas the microporous walls provide a weaker diffusion barrier. Overall, CO_2_ diffuses more rapidly in pretreated sheets with thinner microporous walls, while higher micropore densities provide more space for gas storage, resulting in higher solubility.

### 3.5. Foaming Behavior of PBAT Pretreated Sheets

**Figure 8:**
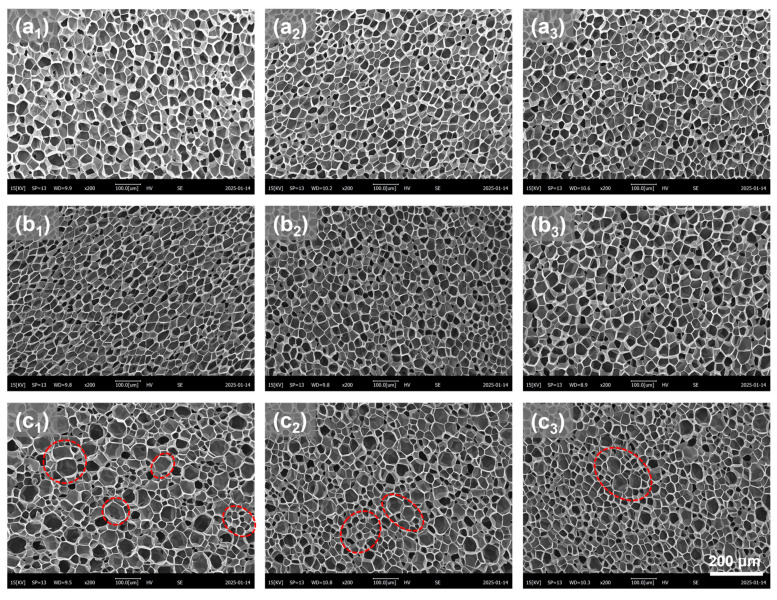
SEM images of foamed PBAT sheets with different microporous structures (red circles show graded cell structures). (**a_1_**–**c_1_**) samples with different micropore sizes of 10 μm, 75 μm and 140 μm at a density of 0.35 g/cm^3^; (**a_2_**–**c_2_**) samples with different micropore sizes of 10 μm, 75 μm and 140 μm at a density of 0.35 g/cm^3^; (**a_3_**–**c_3_**) samples with different micropore sizes of 10 μm, 75 μm and 140 μm at a density of 0.35 g/cm^3^.

**Figure 9:**
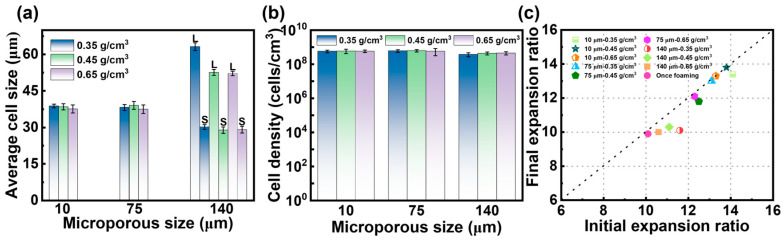
Cell morphology of PBAT foamed sheets with different microporous structures. (**a**) Cell size; (**b**) cell density; (**c**) expansion ratio.

To analyze the impact of the introduced microporous structure on the foaming behavior of PBAT sheets and determine the optimal pretreatment conditions, samples foamed at 100 °C and 18 MPa were selected for microstructural analysis. The SEM images in Figure 8 show the cellular structure after foaming of pretreated PBAT sheets with microporous sizes of 10 μm, 75 μm, and 140 μm. When the micropore sizes were 10 μm and 75 μm, the foamed samples exhibited a uniform cell structure, similar in shape to Figure 4(e_1_–e_3_). However, when the micropore size increased to 140 μm, the foamed samples displayed both large and small cells, resulting in a bimodal cell structure. The corresponding quantitative analysis of the microstructure, including cell size and cell density, is presented in Figure 9a,b. Under identical foaming conditions, the microporous structure introduced during pretreatment affected the cell size of the PBAT foamed samples but did not alter cell density. Specifically, when micropores of 10 μm and 75 μm were introduced through pretreatment, the foamed samples exhibited similar cell size (38 μm) and cell density (5.8 × 10^8^ cells/cm^3^), comparable to those of the foams produced by single foaming of untreated samples. However, when the micropore size reached 140 μm, the post-foaming cell structure became inhomogeneous, with cells differentiating into sizes of 50 μm and 30 μm (indicated by the red dotted line in Figure 8). This suggests that there is a threshold for the size of micropores introduced during pretreatment, beyond which excessively large micropores lead to poor homogenization of foam cells during foaming.

Figure 9c illustrates the effect of the two foaming strategies on the expansion ratio of the foamed samples. The post-foaming expansion ratios of the pretreated PBAT sheets were higher than those of the untreated once-foamed samples, which can be attributed to the gas storage effect of the microporous structure. The increased solubility within the structure provides a greater driving force for cell growth. During the foaming process, variations in the microporous structure and sheet density influence the initial expansion ratio. Notably, the expansion ratio of the foamed samples gradually increased as the micropore size decreased from 140 μm to 10 μm. This may be due to the more uniform distribution of both the microporous and solid structures within the pretreated sheet, which facilitates the expansion of foam cells. Additionally, when the density of the pretreated sheets was reduced, the expansion ratio also increased, likely due to the degree of stretching in the PBAT chain structure. Specifically, when the density of the pretreated sheets was reduced from 0.65 g/cm^3^ to 0.35 g/cm^3^, the expansion ratio increased from 2 to 3.5 times. After pretreatment, the molecular chains underwent further stretching, leading to a foam with a high expansion ratio. PBAT tends to shrink after foaming due to gas exchange and molecular chain relaxation. As shown in Figure 9**c**, the shrinkage after foaming of pretreated sheets with micropore sizes of 10 and 75 μm was lower, with the final expansion ratio remaining similar to the initial one. The highest expansion ratio reached 13.8 times. In contrast, the shrinkage of the pretreated sheet with a micropore size of 140 μm was more pronounced. This could be due to the degree of cell uniformity—the presence of bimodal cells destabilizes the internal structure of the foam, causing faster gas escape and chain relaxation, which results in a significant decrease in the post-stabilization expansion ratio.

### 3.6. Mechanical Properties of PBAT Foams

**Figure 10:**
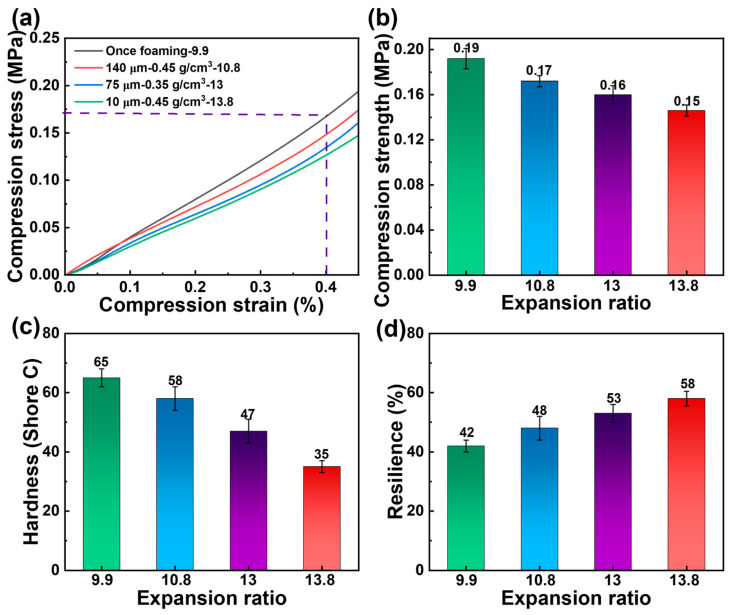
Mechanical properties of PBAT foams. (**a**,**b**) Compression properties, (**c**) hardness, and (**d**) resilience.

Among three pre-treated PBAT foam sheets with varying microporous sizes, the sample with the highest expansion ratio was selected for mechanical characterization (Figure 10). The compression stress of PBAT foams decreases as the expansion ratio increases at the same strain. For untreated, once-foamed samples with an expansion ratio of 9.9 times, the compression strength at 40% strain was 0.19 MPa. In contrast, the expansion ratios of the pre-treated foamed samples were higher than those of the untreated PBAT foam sheets, resulting in a slight decrease in compression strength. As the expansion ratio increased from 10.8 to 13.8, the compression strength of the foamed sheets decreased from 0.17 MPa to 0.15 MPa. However, PBAT foamed samples with varying densities exhibited significant differences in hardness and rebound. As shown in Figure 10c,d, the PBAT foam sheets treated with the pretreatment strategy demonstrated lower hardness and higher resilience when foamed under the same conditions. These findings suggest that the pretreatment strategy enhances foam softness and flexibility, in addition to facilitating rapid saturated foaming.

## 4. Conclusions

In summary, this study systematically examined the effect of PBAT sheet thickness on CO_2_ sorption and diffusion, providing an overview of the sorption kinetics. The dynamic evolution of the cell structure over time during gas diffusion was also explored. The results indicate that while increasing the saturation temperature and pressure enhances the diffusion coefficient, the diffusion process remains challenging in thicker PBAT sheets due to the matrix barrier effect. For a 12 mm thick PBAT sheet, the saturation time can extend up to 9 h. Gas diffusion first reaches saturation in the edge regions and then gradually diffuses towards the core, where the cell structure develops from a no-cell state and progressively unifies toward the edges. Additionally, a new foaming strategy is introduced to shorten the saturation time by introducing different scales of microporous structures into the PBAT matrix through pretreatment. This approach allows CO_2_ to saturate rapidly through the micrometer-sized walls of the cellular pores, diffusing layer by layer into the core of the PBAT matrix. Based on this strategy, the CO_2_ diffusion coefficient for a PBAT thick sheet increased from 1.22 × 10^−9^ m^2^/s to 2.01 × 10^−8^ m^2^/s, and the saturation time was reduced to as little as 2 h. Moreover, the introduction of suitable microporous structures through pretreatment resulted in PBAT foamed sheets with a higher expansion ratio and a similar cell structure to that of untreated PBAT. The pretreated sheets, with a microporous size of 10 μm and a prepared density of 0.45 g/cm^3^, exhibited the best performance after foaming, achieving an expansion ratio of 13.8 times, a hardness as low as 35 C, and a resilience of 58%. Overall, this study establishes a foundation for further research aimed at increasing the CO_2_ diffusion rate in PBAT, which is crucial for enabling rapid and efficient foaming of thick PBAT sheets in industrial applications. The innovative pretreatment strategy not only reduced the saturation time for thick sheets but also improved the foam expansion ratio and resilience, offering a new pathway for producing high-performance flexible biodegradable foams.

## Figures and Tables

**Table 1:** Thermal properties of PBAT samples treated under various temperatures and pressures.

Saturation Condition		T_star_ (°C)	T_mr_ (°C)	ΔH_m_ (W/g)	ΔX (%)
Pressure (MPa)	Temperature (°C)				
10	90	102.8 ± 1.4	38.2 ± 3.1	17.4 ± 0.2	15.3 ± 0.2
	100	107.5 ± 2.5	33.5 ± 2.2	16.2 ± 0.3	14.2 ± 0.3
	110	109.6 ± 1.2	31.4 ± 2.1	15.5 ± 0.2	13.6 ± 0.2
14	90	95.6 ± 1.3	45.4 ± 4.2	16.4 ± 0.3	14.4 ± 0.3
	100	98.3 ± 1.2	42.7 ± 3.1	15.7 ± 0.1	13.8 ± 0.1
	110	99.2 ± 3.1	41.8 ± 3.2	15.2 ± 0.5	13.3 ± 0.4
18	90	92 ± 2.2	49 ± 2.5	14.9 ± 0.1	13.1 ± 0.1
	100	99.1 ± 2.6	41.9 ± 2.8	14.1 ± 0.3	12.4 ± 0.3
	110	101.9 ± 2.7	39.1 ± 1.7	12.3 ± 0.1	10.8 ± 0.1

**Table 2:** Pretreatment parameters for PBAT sheets with different microporous structures.

Saturation Condition		Density (g/cm^3^)	Microporous Size (μm)	Microporous Density (cells/cm^3^)
Pressure (MPa)	Temperature (°C)			
18	70	0.65 ± 0.08	9.2 ± 0.3	2.12 × 10^9^ ± 2.1 × 10^7^
	75	0.45 ± 0.09	10.1 ± 0.8	1.03 × 10^9^ ± 3.6 × 10^7^
	80	0.35 ± 0.06	11.6 ± 1.2	3.34 × 10^8^ ± 2.8 × 10^7^
12	75	0.65 ± 0.03	76.3 ± 4.2	5.34 × 10^6^ ± 5.2 × 10^5^
	80	0.45 ± 0.03	75.6 ± 6.1	6.73 × 10^6^ ± 6.3 × 10^5^
	85	0.35 ± 0.02	72 ± 4.8	7.98 × 10^6^ ± 8.6 × 10^4^
8	90	0.65 ± 0.05	144.3 ± 10.3	9.01 × 10^5^ ± 1.3 × 10^4^
	95	0.45 ± 0.06	140.4 ± 9.6	1.21 × 10^6^ ± 8.6 × 10^4^
	100	0.35 ± 0.07	137.8 ± 17.3	1.54 × 10^6^ ± 7.3 × 10^4^

**Table 3:** Characterization of PBAT sheet foams with different microporous structures.

Microporous Size (μm)	Density (g/cm^3^)	Cell Wall Thickness (μm)	Diffusion Coefficient Ds (m^2^/s)
10	0.35	1.8 ± 0.2	2.011 × 10^−8^ ± 7.331 × 10^−10^
	0.45	2.5 ± 0.1	1.632 × 10^−8^ ± 4.121 × 10^−10^
	0.75	4.5 ± 0.2	1.641 × 10^−8^ ± 1.731 × 10^−9^
75	0.35	13.5 ± 1.6	1.101 × 10^−8^ ± 3.362 × 10^−9^
	0.45	19 ± 2.1	9.942 × 10^−9^ ± 6.221 × 10^−11^
	0.75	33.7 ± 6.2	9.854 × 10^−9^ ± 2.841 × 10^−10^
140	0.35	25.3 ± 1.7	7.763 × 10^−9^ ± 7.344 × 10^−11^
	0.45	35.4 ± 4.1	7.433 × 10^−9^ ± 4.836 × 10^−10^
	0.75	62.9 ± 8.6	7.162 × 10^−9^ ± 5.952 × 10^−10^

## Data Availability

The original contributions presented in this study are included in the article. Further inquiries can be directed to the corresponding author.
